# Prevalence and distribution of acute gastrointestinal illness in the community of China: a population-based face-to-face survey, 2014–2015

**DOI:** 10.1186/s12889-023-15337-z

**Published:** 2023-05-08

**Authors:** Jikai Liu, Baozhang Luo, Yijing Zhou, Xiaochen Ma, Junhua Liang, Xianglai Sang, Le Lyu, Wen Chen, Pengyu Fu, Hong Liu, Shiqi Zhen, Chao Wang, Yangbo Wu, Qiong Huang, Xiaocheng Liang, Guangda Bai, Zhen Lan, Shufang Zhang, Yongning Wu, Ning Li, Yunchang Guo

**Affiliations:** 1 NHC Key Laboratory of Food Safety Risk Assessment, Food Safety Research Unit (2019RU4) of Chinese Academy of Medical Science, China National Center for Food Safety Risk Assessment, Beijing, China; 2 Shanghai Municipal Center for Disease Prevention and Control, Shanghai, China; 3 Jiangsu Provincial Center for Disease Prevention and Control, Nanjing, China; 4 Beijing Center for Disease Prevention and Control, Beijing, China; 5 Guangdong Provincial Center for Disease Control and Prevention, Guangzhou, China; 6 Gansu Provincial Center for Disease Control and Prevention, Lanzhou, China; 7 Jilin Provincial Center for Disease Control and Prevention, Changchun, China; 8 Sichuan Provincial Center for Disease Control and Prevention, Chengdu, China; 9 Henan Provincial Center for Disease Control and Prevention, Zhengzhou, China

**Keywords:** Gastrointestinal disease, Incidence, Care seeking, Community, Diarrhoea, Population survey, China

## Abstract

**Background:**

The true incidence of acute gastrointestinal illness in China is underrecognized by surveillance systems. The aims of this study were to estimate the incidence and prevalence of self-reported AGI in the community of China, and to investigate sociodemographic and epidemiological determinants of AGI.

**Methods:**

We conducted a 12-months cross-sectional population-based survey in eight provinces of China during 2014–2015. The survey determined the prevalence and incidence of acute gastrointestinal illness (AGI) in the total permanent resident population in China according to the census of the population in 2010. The random multilevel population sample was stratified by geographic, population, and socioeconomic status. We used a recommended case definition of AGI, with diarrhea (three loose or watery stools) and/or any vomiting in a four-week recall. A face-to-face survey was conducted by selecting the member in the household with the most recent birthday.

**Results:**

Among 56,704 sampled individuals, 948 (1,134 person-time) fulfilled the case definition; 98.5% reported diarrhea. This corresponds to 2.3% (95% CI:1.9%-2.8%) of an overall standardized four-week prevalence and 0.3 (95% CI: 0.23–0.34) episodes per person-year of annual adjusted incidence rate. There was no significant difference between males and females. The incidence rates were higher among urban residents, and in the spring and summer. In the whole study period, 50% of the cases sought medical care, of which 3.9% were hospitalized and 14.3% provided a biological sample for laboratory identification of the causative agent. Children aged 0–4 and young adults aged 15–24, people living in rural areas and people who traveled frequently had higher prevalence of AGI.

**Conclusion:**

Results showed that AGI represents a substantial burden in China, and will contribute to the estimation of the global burden of AGI. Complemented with data on the etiologies of AGI, these estimates will form the basis to estimate the burden of foodborne diseases in China.

## Introduction

Acute gastrointestinal infections (AGI), including foodborne diseases, are common and cause substantial public health and socioeconomic impact globally. The World Health Organization (WHO) estimated that, in 2010, 22 foodborne enteric agents caused two billion illnesses, over one million deaths, and 78.7 million DALYs [1]. Although enteric disease manifests primarily as self-limiting AGI, appearing as diarrhea or vomiting, more severe disease and even death can occur [2].

Knowledge of the magnitude, distribution, and demographic factors associated with AGI is a requirement for estimating the burden of acute gastroenteritis of foodborne origin, the burden caused by specific pathogens commonly transmitted through foods, and the burden caused by specific foods or food groups [3]. Such evidence is key to define strategies for disease prevention. However, data obtained from public health surveillance does not reflect the true burden of AGI, reflecting any failure in the process between a patient becoming ill and the case being reported: patients often do not seek medical care, samples and laboratory analyses may not be performed, and results may go unregistered [4–6]. In China, a pilot study preformed in six southern provinces between 2010 to 2011 estimated the incidence of AGI [7]. However, it did not include northern and western provinces, and thus was not representative of the whole China. Inspired by this survey, some provinces estimated their own provincial data in the survey or conducted the similar national survey in the next year follow the China CDC’s instruction [8, 9].

The main aims of this study were to estimate the incidence of self-reported AGI in China using a face-to-face method, and to investigate sociodemographic and epidemiological factors as determinants of AGI. Furthermore, we also assessed clinical manifestations and utilization of medical services in cases of AGI. This third nationwide population-based study on AGI covered eight provinces in the period between 2014 and 2015. The estimated AGI incidence also provides an important parameter for estimation the burden of foodborne diseases in China.

## Methods

### Study design and population

We conducted a nationwide population-based retrospective face-to-face survey, implemented over 12 months. The population under study comprised persons resident in the survey site for more than six months; infants born within six months were included. The targeted sample size was calculated according to the residential population of selected provinces, expected incidence, and relative risk (allowed error) with 95% confidence intervals and 20% of the loss rate of follow-up using EPI INFO 3.5.1.

**Fig. 1:**
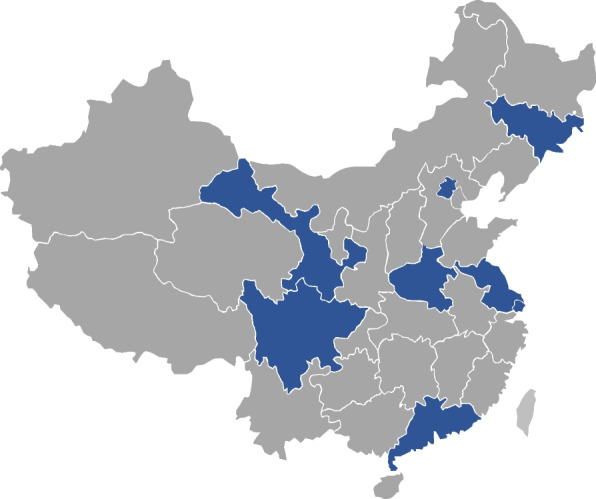
Distribution of survey sites of the population-based retrospective face-to-face acute gastrointestinal disease survey, corresponding to provinces or municipalities in mainland China selected stratified random sampling method

We selected the respondents using a multistage stratified random selection procedure. China has five tiers of local government hierarchy above the household: province, prefecture, county, township, and village. In the first sampling stage, eight provinces or municipalities in China were selected according to geographic, population and socioeconomic status, and food preference by a stratified random sampling method. These province were: Jilin province, Northeast China; Beijing, North China; Shanghai and Jiangsu province, East China; Guangdong province, South China; Henan province, Central China; Gansu province; Northwest China; and Sichuan province; Southwest China (Fig. 1). Simple random sampling (SRS) was used to select three prefecture-level cities or city-level areas within each province in the second stage. In the third stage, two county-level units, including one district and one county-level city, were selected from each prefecture-level city in order to maintain a reasonable ratio between urban and rural areas. SRS selected five villages or towns from each district or county in the fourth stage. In the fifth stage, SRS selected two village-level units from each township unit. In the sixth stage, for each village-level unit, the residential community or village administration supplied the list of numbers for each household. The local community or village general practitioners (GPs) are the interviewers who used SRS selected eight households each month from the list; households that were previously selected and interviewed would not be selected again. GPs selected 96 households per village or town for twelve months by SRS.

Before the survey, all the involved provincial and cities’ CDC experts took a 3-days training to explain the methods and content of the questionnaire. City level CDC experts gave the GPs in the communities in the districts which they selected a similar training but in local dialects and customs.

In the selected communities or villages, GPs acquainted with the residents entirely and have their contact information which is necessary during the daily routine work. Before interview, To allow for the maximum number of people to be contacted, they contacted the household by phone, messaging app or face-to-face to book the time to meet when most of the residents in the household. Contact of the selected households was attempted up to three times. The GPs made an appointment with the members of the household during off-duty hours if some of them were working in the daytime. The household was calculated as a loss to follow-up if the household remained uncontacted up to three times or refused to be interviewed without a neighboring replacement. Then the GPs used standard and unified printed empty questionnaires visited household. The interviewer determined if the household contained two or more adults, and then asked the adult household member who had the most recent birthday [10, 11] in a face-to-face way. If the household was successful interviewed, the rest of the members wouldn’t be asked, and household was interviewed only once. Study participants were enrolled from April 2014 to March 2015. All the interviews were conducted in Chinese with local accent, including Cantonese and other dialects to make understandable by the interviewers and the questions were asked in a colloquial but in line with the correct meaning of those AGI symptoms.

The representativeness of the sample was improved by weighing. These weights were generated to avoid deviation of the target population to the whole Chinese population based on data from 2010 (6^th^) National population census in China [12].

The questionnaire collected self-reported socio-demographic information on the respondent (gender, age, education, occupation of the main earner, family income and jurisdiction), recent travelling history, information on the etiology of infection, AGI symptoms, related health care-seeking behaviors, treatment (available verification like prescriptions, medication boxes or pill strips are suggested), eventual absence from work associated with illness and direct/indirect expenses.

### Case definition

To ensure that the case definition was simple and applicable, as well as comparability with the results from other surveys conducted globally, we used the standard case definition advocated by the international collaboration on enteric disease ‘Burden of Illness’ Studies [13]. AGI was defined as *having had at least three episodes of loose stools or any vomiting in any 24-h period*. Participants who reported having fewer than three loose stools in a 24-h period were not counted as a case. The same participants who reported another AGI after 7 days with in 28 days before the interview counted as having the AGI twice. Any AGI symptoms caused by a non-infectious gastrointestinal illness like chronic gastrointestinal diseases (Crohn’s disease, ulcerative colitis, stomach cancer, and intestinal tumors, irritable bowel or coeliac disease), or alcohol or drugs, or normal physiological phenomena like pregnancy and menstruation were also excluded.

### Data analysis

EpiData version 3.1 (EpiData Association, Odense M, Denmark) was used for data collection. All data were weighted by sampling frame and adjusted by age, gender and residency based on the sixth national population census data in 2010. The analysis was performed using SAS 9.4 (SAS Institute Inc., Cary, NC, USA).

Four-week period prevalence (I4 wk.) and incidence proportion (expressed in %) were calculated, along with 95% confidence intervals (CIs). The annual incidence (I annual) was calculated as I annual = I4 wk. × (365/28) and expressed in terms of episodes/person per year [13]. Qualitative data were described using percentages or proportions, and quantitative data were statistically described using mean and 95% confidence intervals. Values of *P* < 0.05 were considered statistically significant. The aim of calculation the four-week period prevalence is to estimate the annual episodes of the AGI. The null hypothesis of no association between prevalence of disease and age group, cultural group, total per capita household income level, and highest education level was tested using ${x}^{2}$ test. We measured the association between disease determinants as explanatory variables and the defined AGI cases as the outcome variable, defined as odds ratios (ORs), using multivariable logistic regression. For comparison of average means between age groups and gender, the two-tailed *P*-value from linear regression was used.

### Ethical approval

Ethical approvals were granted by China National Center for Food Safety Risk Assessment’s Ethical Board. All data collected including the names, addresses, and telephone numbers of the participant were kept confidential. Each participant was asked to read and sign a consent form that informed about the purpose of the survey before the questionnaire was administered.

## Results

### Sample and response rates

In total, 58,320 interviews were completed in the period between April 2014 to March 2015. Among these, 56,704 (97%) valid responses were obtained.

### AGI occurrence

**Fig. 2:**
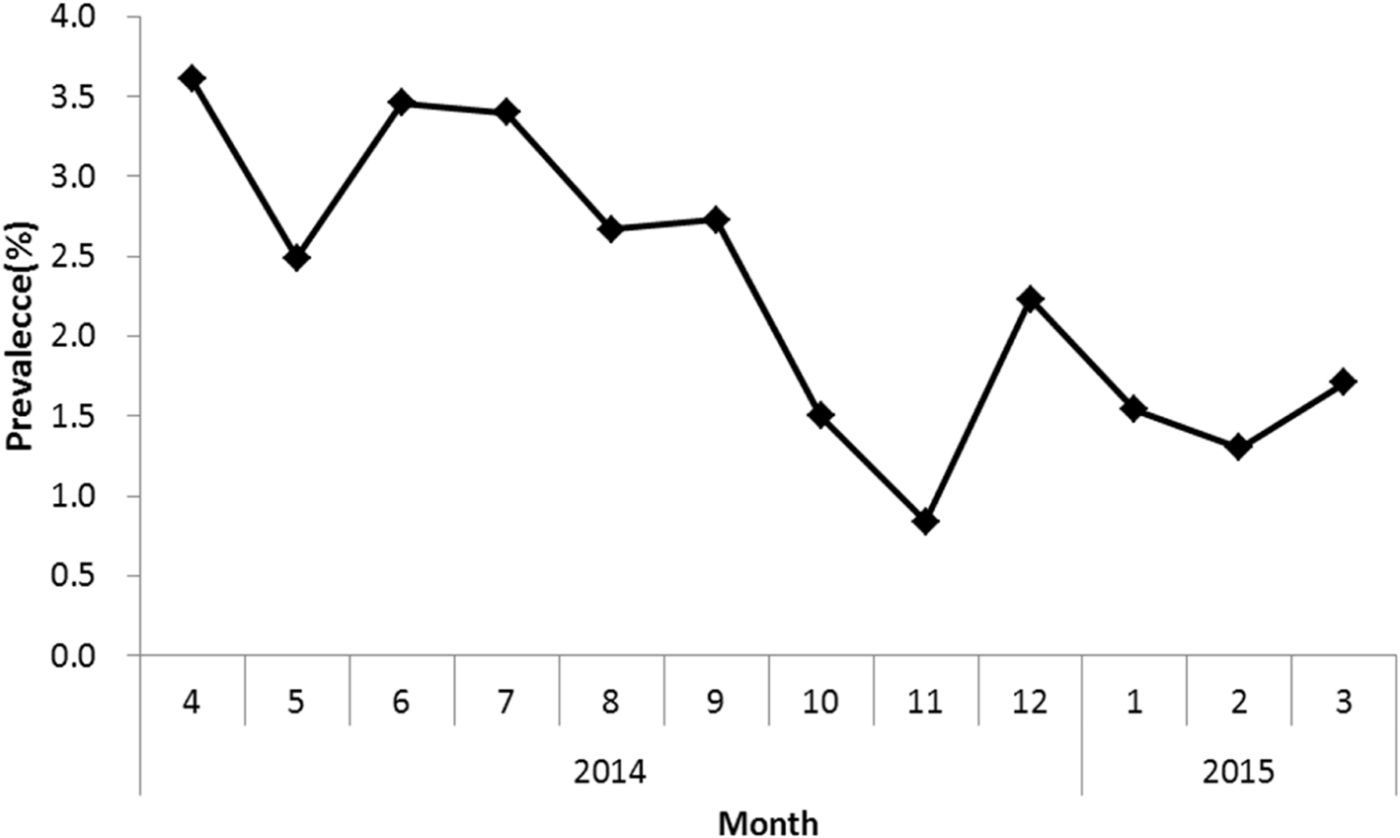
Prevalence of acute gastroenteritis in the overall population by month, 2014–2015

At least one episode of acute gastroenteritis was reported by 2.3% (95% CI: 1.9–2.8%) of respondents in the four weeks before the interview, corresponding to a rate of 0.3 (95% CI: 0.23–0.3) episodes per person per year. The prevalence was higher in summer months (Fig. 2). The mean duration of illness (the AGI related symptoms) was 11.3 h (95% CI: 3.5–14.5). Half of the respondents seeked medical care (49.8%; 95% CI: 43.9–55.6). Nearly half (47.0%; 95% CI: 42.4–51.6%) of the patients who seeked medical care were taking some type of medication because of AGI; 17.6% (96% CI: 14.4–21.4%) reported taking anti-diarrheal medication.

**Fig. 3:**
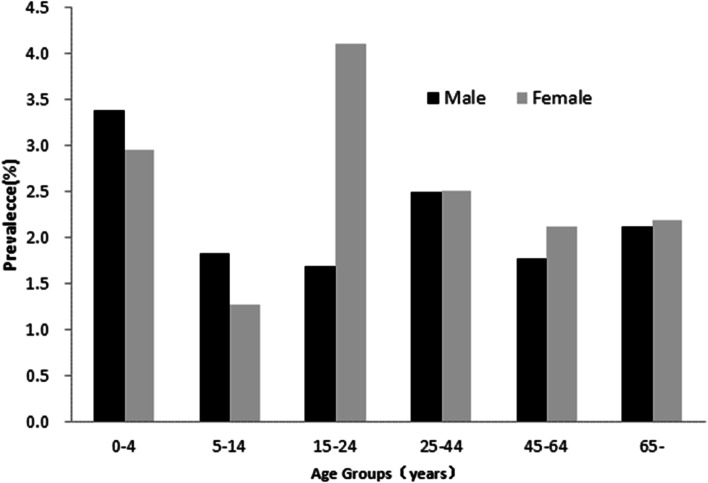
Prevalence of acute gastrointests in mainland China, by age and gender

Although there were some differences in the prevalence of AGI in some age groups, particularly in the population 15–24 years of age (Fig. 3), results showed there were no significant differences in the incidence of AGI between males and females. The incidence was higher among children below 5 years (Fig. 3). Results of multivariate analysis showed that occupation of the main earner, and traveling history were significantly associated with acute gastroenteritis (Table 1).

**Table 1:** Incidence estimates and determinants for acute gastrointestinal illness (AGI) in China, 2014–2015 (weighted) (*n* = 56,704). Statistically significant values are highlighted in bold

Determinants	Sample size	No. of AGI cases	four-week period prevalences	Annual incidence^b^	95% CI^a^	OR	95% CI	*P* value
		(unweighted)						
**Total**	56,704	1,134	2.16	0.28	(0.23–0.34)	-	-	-
**Gender**								
Male (*n* = 28,047)	28,047	547	1.96	0.26	(0.19–0.32)	1.07	0.94–1.22	0.298
Female (*n* = 28,657)	28,657	587	2.36	0.31	(0.22–0.40)	Ref	Ref	Ref
**Age**								
< 5	1,493	44	2.93	0.38	(0.22,0.55)	**0.61**	**(0.44,0.85)**	**0.003**
5–14	2,777	55	1.46	0.19	(0.10,0.30)	1.03	(0.76,1.41)	0.844
15–24	3,431	66	2.49	0.33	(0.17,0.50)	1.06	(0.79,1.41)	0.713
25–44	14,870	318	2.34	0.3	(0.21,0.40)	Ref	Ref	Ref
45–64	22,323	426	1.8	0.23	(0.17,0.30)	1.15	(0.98,1.35)	0.085
≥ 65	11,810	225	2	0.26	(0.17,0.36)	1.12	(0.93,1.35)	0.233
**Ethnicity**								
Han	55,210	1098	2.22	0.29	(0.23,0.35)	1.127	(0.77,1.65)	0.537
Other	1,494	36	0.58	0.08	(0.01,0.15)	Ref	Ref	Ref
**Geographical**								
North	28,075	555	1.8	0.23	(0.15,0.32)	1.12	(0.98–1.27)	0.097
South	28,629	579	2.4	0.31	(0.24,0.38)	Ref	Ref	Ref
**Residence**								
Urban	26,178	505	2.65	0.23	(0.15,0.32)	1	(0.88, 1.14)	0.982
Rural	30,526	629	1.81	0.31	(0.24,0.38)	Ref	Ref	Ref
**Education**								
Preschool children	1900	49	2.41	0.31	(0.18,0.45)	**0.72**	**(0.52,1.00)**	**0.052**
Illiterate	4065	96	2.24	0.29	(0.12,0.48)	0.95	(0.72,1.25)	0.696
Primary school	13,301	286	1.89	0.25	(0.17,0.33)	1.01	(0.82,1.24)	0.913
Middle school	18,388	340	1.74	0.23	(0.14,0.33)	**1.22**	**(1.00,1.49)**	**0.055**
High school	10,579	196	2.25	0.29	(0.16,0.43)	1.16	(0.93,1.45)	0.19
Undergraduate and above	8,471	167	3.14	0.41	(0.27,0.55)	Ref	Ref	Ref
**Occupation**								
While collar job	7,402	175	3.49	0.45	(0.27,0.66)	Ref	Ref	Ref
Blue collar job	17,624	377	2.14	0.28	(0.17,0.38)	**0.82**	**(0.68,1.00)**	**0.053**
Others	31,678	582	1.85	0.24	(0.19,0.30)	1.08	(0.94,1.25)	0.281
**Per capita household income**								
< 20,000 RMB/person	28,822	617	1.82	0.24	(0.17,0.31)	Ref	Ref	Ref
20,000–50,000RMB/person	17,347	351	3.01	0.39	(0.27,0.53)	1.01	(0.88,1.17)	0.864
> 50,000 RMB/person	5,270	92	2.16	0.28	(0.14,0.43)	1.17	(0.92,1.48)	0.205
Non-reported	5,265	74	1.95	0.25	(0.12,0.38)	**1.38**	**(1.07,1.78)**	**0.015**
**No. of persons in household**								
1	7,382	122	2.53	0.33	(0.17,0.53)	Ref	Ref	Ref
2	19,689	426	2.75	0.36	(0.24,0.48)	**0.8**	**(0.64,1.00)**	**0.046**
3	16,188	317	1.93	0.25	(0.16,0.35)	0.88	(0.70,1.10)	0.27
≥ 4	13,445	269	1.94	0.25	(0.17,0.34)	0.88	(0.70,1.12)	0.3
**Travel within two weeks**								
Yes	1,001	36	6.81	0.89	(0.39,1.46)	Ref	Ref	Ref
No	55,701	1092	2.08	0.27	(0.22,0.33)	**0.41**	**(0.30,0.50)**	** < 0.001**
Non-reported	1	-	-	-	-	-	-	
**Province**								
Beijing	9,885	131	0.73	0.1	(0.05,0.15)	Ref	Ref	Ref
Jilin	5,906	167	3.04	0.4	(0.24,0.56)	**0.48**	**(0.37, 0.62)**	** < 0.001**
Shanghai	8,407	149	1.62	0.21	(0.14,0.28)	**0.73**	**(0.57, 0.95)**	**0.018**
Jiangsu	6,948	187	3.58	0.47	(0.31,0.63)	**0.47**	**(0.37, 0.59)**	** < 0.001**
Henan	5,433	63	1.14	0.15	(0.07,0.22)	1.06	(0.77, 1.46)	0.717
Guangdong	5,745	108	1.99	0.26	(0.16,0.36)	**0.66**	**(0.50, 0.86)**	**0.003**
Sichuan	7,529	135	2.25	0.29	(0.20,0.39)	**0.72**	**(0.55, 0.94)**	**0.014**
Gansu	6,851	194	3.15	0.41	(0.21,0.64)	**0.54**	**(0.42, 0.69)**	** < 0.001**

^a^*CI* Confidence interval

^b^Annual incidence: specified as episodes per person-year

For 17.4% (95% CI: 14.2–21.2%) of those with AGI, they or a member of their family took time off work due to the diarrhea episode. The median number of days off was two, and the mean was 2.7 days.

### Socio-demographic characteristics of AGI

Estimates of the four-week period and annual incidence of AGI in China with specific demographic characteristics are presented in Table 1. There was no significant difference in the incidence of AGI between males and females (0.26 vs 0.31). Except for the traits of age groups mentioned above, the overall incidence of AGI was high in Han nationality than in other nationality (0.29 vs 0.08, *P* = 0.537). The prevalence of AGI in the north of China was slightly lower than the rate in the south (0.23 vs 0.21, *P* = 0.097). There was no significant difference in the incidence of AGI between Urban and Rural residence and per capita household income. The tendency of incidence seemed highest in preschool children and declined with the increase of educational levels; however differences were not significant. There was a borderline significant difference in incidence of AGI between participants with blue collar jobs and with white collar jobs (0.28 vs 0.45 *p* = 0.053). The incidence of AGI was significantly higher in participants who had travelled within two weeks than in those who had not (0.89 vs 0.27). Respondents living in a household with two persons were significantly less likely to have experienced AGI than individuals living alone. The prevalence of AGI varied by provinces, with the highest in Gansu (0.41, *P* < 0.001), and the lowest in Beijing (0.10, *P* < 0.001).

#### Symptoms, healthcare, and impact of AGI

Among the 1,134 cases, 1,122 (98.9%) reported suffering from diarrhea, 238 (21%) reported suffering from vomiting and 226 (19.9%) cases experienced both symptoms. Of the 1,122 cases with diarrhea, 16 (1.4%) had bloody diarrhea. Respiratory symptoms were reported by 10.4% of all the cases, usually not associated with intestinal disease. The information on the duration of illness, presence of concurrent symptoms and recourse to healthcare is reported in Table 2. The average duration of illness was 46.4 h. Children in the 0–4 year group had a significantly higher fever rate (39.5%) than other age groups (*p* < 0.001). On the worst day of symptoms, cases reported diarrhea an average of 4.2 times (range 3–16 times) and vomiting an average of 2.4 times (range 1–11 times).

**Table 2:** Self-reported proportions and average means for associated factors, care seeking and treatment of acute gastrointestinal illness cases in China, by age and gender (*n* = 948)

	Age group (years)							*P *value for age	Male	Female	*P* value for gender
	Total	0–4	5–14	15–24	25–44	45–64	65-				
Diarrhea (%)	98.94	97.67	97.87	98.28	98.87	99.42	98.94	0.283	99.11	98.79	0.624
Vomiting (≥ 1 times/day) (%)	20.95	39.53	36.17	22.41	22.26	17.44	17.02	** < 0.001**	18.4	23.28	0.066
Bloody diarrhea (%)	1.39	2.38	0	0	2.29	0.58	2.15	0.665	0.89	1.84	0.225
Fever (> 38 °C) (%)	11.85	39.53	29.79	6.9	10.94	9.88	7.45	** < 0.001**	11.97	11.74	0.912
Respiratory symptoms (%)	10.36	27.91	12.5	6.9	8.3	10.17	10.11	0.173	11.5	9.31	0.27
Stool sample (%)	11.88	15.63	3.57	20	8.65	11.11	17.74	0.408	8.7	14.67	0.09
Outpatients (%)	36.65	74.42	59.57	34.48	39.62	28.86	32.98	** < 0.001**	35.7	37.53	0.561
Hospitalized (%)	4.02	0	0	5	4.76	4	6.35	0.059	1.84	5.95	0.066
Work absenteeism (%)	13.21	9.3	14.89	15.52	20.08	11.5	6.38	**0.001**	12.22	14.11	0.394
Medication taken (%)	78.69	95.35	85.11	72.41	75.85	76.61	82.98	0.838	78.44	78.9	0.863
Medication prescribed (%)	42.72	68.29	57.5	42.86	47.76	35.5	37.82	** < 0.001**	42.78	42.67	0.978
Diarrhea medication (%)	45.6	69.23	35.56	34.48	45.95	43.15	50.54	0.557	46.22	45.04	0.719
Antibiotics medication (%)	44.75	25.64	66.67	44.83	46.54	44.35	41.62	0.315	44.65	44.83	0.954
Painkiller medication (%)	5.12	5.13	8.7	8.62	6.59	2.69	5.46	0.089	5.52	4.76	0.604
Antipyretic medication (%)	2.84	15.79	6.52	5.17	3.49	1.2	0.55	** < 0.001**	2.53	3.11	0.6
Mean duration of using medication (hours)	46.43	49.95	35.92	31.1	45.63	42.47	n.a	0.497	43.56	49.03	0.061
Mean duration of symptoms (hours)	42.19	50.11	37.52	35.19	41.39	38.49	n.a	0.068	40.92	43.37	0.398
Mean duration of hospitalization (hours)	121.71	n.a	n.a	72	115.2	156	108	0.814	56	139.64	0.151
Mean duration of work absenteeism (days)	2.47	n.a	1.84	1.44	2.1	3.13	n.a	**0.001**	2.36	2.56	0.572
Mean duration before seeing doctor (hours)	15.36	15.56	12.61	10.1	16.1	13.56	19.82	0.218	15.5	15.24	0.897

The healthcare-seeking behaviors of AGI cases, medication had been taken because of the AGI by cases, and the source of the medicine are reported in Table 3. 36.7% of all the cases took outpatient services, while 4% were hospitalized. Outpatient services-seeking behaviors varied by age; the percentage of service-taking for AGI was highest (74.4%) in children aged < 5 years. 11.9% of all the cases reported providing a stool sample for laboratory testing. In total, 78.7% of all the cases took medication for AGI. Nearly half (44.8%) of all cases have taken antibiotics, while 5.1% reported taking pain killers and 2.8% antipyretics. Only 42.7% reported that the medication was prescribed by a doctor.

**Table 3:** Distribution of medical institutions utilized by acute gastroenteritis cases

**Hospitals**	Outpatients		Hospitalized	
	*n* = 346	%	*n* = 14	%
**Grades of hospital**				
Tertiary hospital	26	7.51	0	0.00
Secondary hospital	48	13.87	8	57.14
Primary hospital	81	23.41	5	35.71
Other hospital	191	55.20	1	7.14
**Types of hospital**				
General hospital	113	32.66	10	71.43
Children's hospital	4	1.16	0	0.00
Chinese medicine hospital	12	3.47	2	14.29
Other hospital	217	62.72	2	14.29
**Classification of hospital**				
Provincial and ministerial hospital	6	1.73	0	0.00
City hospital	31	8.96	1	7.14
County hospital	28	8.09	4	28.57
Community health center	110	31.79	6	42.86
Health service station	130	37.57	0	0.00
Private hospital	9	2.60	3	21.43
Individual clinic	31	8.96	0	0.00
Other hospital	1	0.29	0	0.00

#### Self-reported suspect causes of AGI

Among the positive individuals who reported the cause of AGI, 59.3% reported that their AGI could be food related. Of the food related individuals, 17.5% thought it was due to meat and its products (pig meat, beef and mutton), 13.3% thought it was due to fruit and its products, 12.9% thought it was due to vegetable and its products. The distribution of other food categories was scattered. Of the individuals who could recall the food source, 38.7% from home, 12.9% from fast-food restaurant or snack bar, 11.7% from street stalls.

## Discussion

We estimated the magnitude and distribution of AGI in the community in China using a large-scale population-based study. Our results showed that the prevalence of AGI in the population was 2.3%, corresponding to an incidence of 0.3 episodes per person per year. In other words, our study suggests that one in every three Chinese residents suffers from AGI every year. This was the first survey provide nationwide representative estimates of the prevalence and incidence of AGI in China.

Applying the synchronized interviewing cycle and the same questionnaire, this survey covered not only north and south representative provinces in China, but also provinces representing the northwest (Gansu province) and southwest (Sichuan province), thus providing national representative data on the incidence and distribution of AGI in the population. We applied a face-to-face methodology like what has been used in other studies [14, 15]. Similar to those studies, we achieved a high average response rate of 97.2% when compared to telephone-administered surveys conducted in most developed countries. The high response rate was not only determined by the face-to-face interviewing, but also greatly benefited from cooperative street residents' committee from selected residential district or villages.

### Seasonality and geography

The results showed a seasonal distribution of AGI during the study period that is in line with the normally expectation- of high incidence of AGI in summer and low incidence in winter. The data showed its peak in spring and summer, and lower incidence in autumn and late winter, but relative high in early winter. The variation is likely to reflect the seasonal variation in infections with viral enteric pathogens in colder months, as seen in other studies [2, 16–18]. China is so large that temperatures and other meteorological factors typical of different seasons vary by province during the same period. For example, when the southern region is in summer, the northern region is in early spring, and in topography descends from the west to the east. As a result, the seasonality of AGI of bacterial origin is not synchronized. An analysis stratified by province and season would be needed to clarify such variations.

### Determinants

We did not observe significant differences in the likelihood of having AGI in individuals with different levels of education, with different levels of income, or living in urbanized or rural areas. Travelling within two weeks was a significant risk factor, which is in accordance with what seen elsewhere [19].

### Healthcare utilization

Care seeking behavior and utilization of healthcare services was high in all age groups. Half of surveyed individuals that reported AGI went to primary and lower health care institutions for medical care, which represents a higher rate of seeking medical care than estimated by other countries such as the United States (19%) [19], Ireland (19.5%) [20], Canada (20.4%) [21], Australia (19.5%) [22] and Denmark (12%) [16], Cuba (17.1%-38.1%) [23] and Argentina (26%) [14]. It remains to be investigated if this disproportion is caused by the attention to disease, the attitude towards medicine., different medical insurance systems or differences in healthcare systems. The collection rate of biological samples in this survey was 14.3%, which is close to Canada (14.4%) [21] and Ireland (14.9%) [20], but lower than Australia (18.4%) [22] and the United States (21.1%) [19]. These results showed that the attention of hospitals in China to investigating the causes of AGI still needs to be improved and suggest that the central and local governments should increase awareness and investment to improve the sampling rate of patients' and the rate of laboratory diagnostics, to better grasp the etiology of acute gastroenteritis.

The hospitalization rate of AGI in our survey was 7.9%, suggesting a high proportion of severe cases of AGI in China. This index was not included in the previous domestic survey [7].

Nearly 80% of cases were treated with drugs, among which 62% use antibiotics, a much higher proportion estimated in other countries (US: 8.3%, Ireland: 5.6%, Canada: 3.8%, Australia: 3.6%, Italy: 6.5% Argentina: 7%, Cuba: 6.5–18.9%) [2, 14, 19–21]. Overuse or misuse of antimicrobials may lead to antimicrobial resistance, an important public health concern globally. These estimates suggest that there is still a long way to go to popularize the legal knowledge of the scientific use of antibiotics in China.

### Domestic comparison

The estimated incidence of AGI in China of 0.28 (95%CI: 0.23–0.34) episodes per person was lower than the rate of 0.56 (95% CI: 0.56–0.57) episodes per person per year estimated by the previous survey conducted between 2010 to 2011 [7]. However, they were like the estimation of 0.31 episodes per person per year calculated by reviewing scientific literature [24]. The differences in the estimates of the different surveys may be explained by several factors. First, differences in the selection of provinces in the survey design, since China has great population diversity, which may also be reflected in diarrhea incidence and risk factors. All the provinces selected by the previous survey were southern provinces. On the contrary, our study took place in eight provinces that represent all the traditional seven regions of China [25, 26]. Second, in the past several years, the Chinese government has continuously improved the food safety regulation systems, developed innovative regulation mechanisms, established a structure for developing food safety standards and successfully dealt with intentional food safety issues [27]. Risk communication was also enhanced. As examples, many ministries and departments jointly organized the Food Safety Awareness Week and Open Day. Several activities based on data generated from greatly improved foodborne disease surveillance since 2011 were also implemented. In 2013 alone, 120,000 supervision staff, more than 4,000 experts and scholars, and 35 million employees participated in activities. Hundreds of media issues nearly 20,000 news reports and over 300, 000 micro-blogging topics [27]. These activities have also had an impact on public awareness and engagement in food safety practices. The overall food safety status has improved steadily and may be reflected in the burden of diarrheal diseases.

### International comparison

Several cross-sectional surveys have been conducted in other countries to estimate the prevalence and distribution of AGI. In America [15, 28–38], Europe [2, 16–18, 39–44], Oceania [45, 46], Asia [47] and Africa [48].

The estimated incidence of AGI in China is comparable to similar retrospective studies conducted in Sweden and France, which reported 0.31 and 0.33 episodes of AGI each year, respectively, despite slightly different case definitions and recall periods. It is also similar to estimates of prospective follow-up studies in The Netherlands (0.28) (ranging from 0.42 to 1.66 episodes/person-year) [49]. However, it is lower than observations in almost all the similar cross-sectional studies conducted in other countries or regions, no matter what interviewing methods they used, and higher than rate from England and Wales (0.19) [50]. Nevertheless, comparisons between countries need to consider the varying case definitions, interviewing and sampling methods, and general differences in populations.

Our study used criterion for the identification of AGI recommended by an international collaboration focusing on burden of foodborne illnesses [51]. However, most studies, unlike ours, used a telephone survey; some of them used random digit dialing techniques or modified version to select participants [2, 16, 18, 47]. Other possible explanations for the differences in AGI incidence include cultural aspects and likelihood to answer questions openly. Chinese people are not used to talk about their health status in front of strangers, no matter if in public or in private, especially in big cities like Beijing and Shanghai. Furthermore, our survey had a very high response rate. With the help of social workers in resident's committees or villager's committees, almost all the selected household participated, and we had a response rate of 97%. In contrast, the response rate in other countries was below 70%, but there is a possibility that people who have AGI recently are more inclined to respond. Other explanations for differences in reported incidence of diarrhea may include differences in risk factors such as food consumption and preparation habits, food contamination, or environmental factors. Furthermore, 59.3% of individuals reported that consuming suspected food was the cause of their AGI which is much higher than the proportion in Hong Kong (45.0%) [47] and Ireland (18.5%) [20]. The most self-reported suspected food categories(meat, fruit and vegetable) was correlated with the tendency of foodborne outbreaks [52] and diseases surveillance [53] in the past 10 years.

### Limitations and strengths

In this survey, the samples were weighted and standardized according to the result of 6^th^ national population census in 2010 with the structure of age and gender directly, to make the survey sample representative. A sample weight was assigned to each sample person. Sample weights were considered measures of the number of people represented by the particular sample person. However, there are still some biases and lack of consideration in the survey: (i) We did not consider design effects (DEFF) when designing the survey, so the results may be limited to the sample and cannot be extrapolated to the entire population. However, standardizing the sample and overestimating the loss rate of follow-up and relative risk could neutralize the bias of ignoring the DEFF; (ii) Inevitably, young and middle aged subgroup whose residency is in rural area but worked in the cities were not sampled according to the characteristics of the census, the occurrence of left-behind elderly and children in the sample were higher than reality. We generated the weights to adjust the target population. However, the adjustment works only if there is someone were sampled in the subgroup in someplace, for those didn’t, there is no adjustment which might result in bias. (iii) In addition, the selection of survey sites failed to give full consideration to China's western minority areas, such as Tibet and Xinjiang. At the same time, some groups of group-life accommodation (such as inpatients in hospitals, elderly people in nursing homes, students in long-term accommodation, prisoners in prisons and officers and soldiers in barracks) were not covered. This might have introduced selection bias. (iv) No question about food preparation preferences and personal hygiene were involved in the questionnaire, and further research should examine these so that appropriate prevention measures can be put in place. (v) Our study suffered from the limitations of all cross-sectional surveys. Its results reflect only real time data from the survey sample and is difficult to make causal inferences. (vi) As a cross-sectional survey, this study should use prevalence rate ratios instead of odds ratios. Furthermore, to describe those AGI-like acute diseases, we should also choose the prevalence rate ratios, since using odds rations might amplifying the numbers.

### Impact

Despite these limitations mentioned above, our estimates provide evidence on the incidence of AGI in the population of mainland China in seven regions for the first time. Due to underdiagnosis and underreporting, such data are not available from public health surveillance. However, it is crucial to demonstrate the true burden of diarrheal diseases in the population. This burden is not only of a health nature, but also social and economic. For example, our estimates demonstrated that at least 17% of respondents or caretakers with AGI loss a mean 3 days of work due to the illness. If extrapolated to the population, this equates to approximately 1.5 million working days loss due to AGI annually. Together with healthcare costs, this represents a substantial burden nationally. When combined with evidence on the contribution of different causative agents for the overall incidence of AGI, these estimates form the basis to estimate the burden of foodborne diseases in China.

## Data Availability

All data generated or analyzed during this study are included in this article.

## Abbreviations

AGI: Acute gastrointestinal illness
SRS: Simple random sampling
CDC: Centers for Disease Control and Prevention
CI: Confidence interval
REF: References
OR: Odds ratio
SAS: Statistics Analysis Systems
